# Enhanced Adsorption Performance Cross-Linked Chitosan/*Citrus reticulata* Peel Waste Composites as Low-Cost and Green Bio-Adsorbents: Kinetic, Equilibrium Isotherm, and Thermodynamic Studies

**DOI:** 10.3390/polym15153246

**Published:** 2023-07-30

**Authors:** Deniz Akin Sahbaz

**Affiliations:** Department of Chemical Engineering, Faculty of Engineering, Pamukkale University, Denizli 20070, Turkey; dsahbaz@pau.edu.tr; Tel.: +90-258-296-3094

**Keywords:** *Citrus reticulata* peel waste, chitosan, Congo red, green adsorbents

## Abstract

This study revealed the synthesis of cross-linked chitosan/*Citrus reticulata* peel waste (C/CRPW) composites that could be used as low-cost and green bio-adsorbents for the removal of Congo red (CR) dye from aqueous solutions. C/CRPW composites containing different amounts of *Citrus reticulata* peel waste (CRPW) and chitosan were prepared and cross-linked with glutaraldehyde. The composites were characterized by FESEM, EDS, FTIR, XRD, BET, and zeta potential measurements. The C/CRPW composites as a new type of bio-adsorbents displayed superior adsorption capability toward anionic CR molecules, and the adsorption capacities increased with the incorporation of CRPW. Effects of different ambient conditions, such as contact time, pH, adsorbent dosage, initial adsorbate concentration, and temperature, were fully studied. The conditions which obtained 43.57 mg/g of the highest adsorption capacity were conducted at pH 4 with an initial concentration of 100 mg/L, adsorbent dosage of 2.0 g/L, and contact time of 24 h at 328 K. The adsorption data was found to follow the pseudo-second-order kinetic model and the Freundlich isotherm model. According to the findings of this investigation, it was observed that the C/CRWP composites could be used as adsorbents due to their advantages, including the simple preparation process, being environmentally friendly, renewable, efficient, and low-cost.

## 1. Introduction

Dye pollution in the aquatic water system is a serious environmental problem because of the decrease in the photosynthetic activity of water streams and the equilibrium disruption of the aquatic environment. Most synthetic dyes found in many industrial wastewater effluents are also toxic and carcinogenic to both human and animal health at a very low percentage of concentration. Various physical, physicochemical, chemical, and biological treatment processes have been used to remove dyes from wastewater, including adsorption [1], membrane separation [2], coagulation–flocculation [3], ion exchange [4], and ozonation [5]. Among these techniques, adsorption has superior advantages, such as low investment and operational cost, simple design, easy operation, high effectiveness, and so on, for removing dyes.

Congo red (CR) [1-naphthalenesulfonic acid, 3,3′-(4,4′-biphenylenebis (azo)) bis(4-amino-) disodium salt] is an anionic diazo dye [6] used in several industries, such as paper, plastic, leather, textiles, etc., for coloring their final products. Wastewater containing CR dye is a kind of threatening wastewater because of the difficulty of its degradation. In addition, a carcinogen product, such as benzidine, is formed because of its decomposition under anaerobic conditions [7]. In this present study, CR is chosen as an anionic dye for the adsorption process due to its chemical structure, environmental concern, and potential toxicity to humans. Many adsorbents, such as carbonized leonardite [8], nickel-based materials [7], MIL-88A [9], MXene–carbon foam hybrid aerogel [10], and polycrystalline α-Fe_2_O_3_ nanoparticles [11], have been reported in the literature for the adsorption of CR dye. However, most of the commercial adsorbents used in the treatment of dye effluent are not economically viable and some are not technically efficient.

Agricultural waste materials have attracted more attention to be used as adsorbents for the removal of contaminants in aqueous solutions due to their viable properties, such as being eco-friendly, renewable, their biodegradable nature, being inexpensive, available in abundance, and the easy-to-obtain composites with them. Furthermore, agricultural wastes have various functional groups which enhance their chemical reactivity and are responsible for the removal of various water pollutants. Some researchers have studied the feasibility of using agricultural solid waste, such as coir pith [12], potato plant [13], durian peel [14], grapefruit peel [15], mandarin orange peels [16], citrus limetta peel [17], orange peel [18], kiwi peel [19], etc., as adsorbents for the removal of dyes and/or heavy metals from wastewater. 

Mandarin (*Citrus reticulata*) peel waste, an agricultural waste from peels that accounts for approximately 30% of the mass of mandarin fruit, is obtained as by-products from the food industry and juice companies, besides household waste [20]. It is estimated that around 110–120 million tons of citrus waste are produced annually worldwide. The dumping of this waste in landfills or marine environments results in nitrogen deficiency during microorganic activities and a significant increase in the levels of biological oxygen demand and chemical oxygen demand [21]. Hence, the reusability of this waste in different areas is significant due to increased environmental damage. The *Citrus reticulata* peel waste (CRPW), abundantly available in Mediterranean countries, Brazil, Japan, Argentina, the United States, and Australia, can be used as an effective adsorbent for the removal and recovery of dyes and heavy metals from wastewater because of the various functional groups, such as hydroxyl, carboxyl, phenolic, and amino [22]. In this study, CRPW was utilized as an alternative material that offers a high affinity for dye molecules to synthesize polymeric composites as bio-adsorbents. 

Chitosan, which has been obtained from the alkaline hydrolysis of chitin, is one of the most popular adsorbents for various adsorption processes due to its high absorptivity and affinity for environmental contaminants, biodegradability, biocompatibility, nontoxicity, hydrophilicity, physicochemical characteristics, high chemical reactivity, and hydrophilicity [23]. Because of its significant advantages, chitosan has positive feedback from many researchers employing chitosan as an adsorbent. However, chitosan has disadvantages, such as poor mechanical strength, thermal instability, and low surface area. In recent studies, it has been observed that this problem has been largely overcome with the use of chitosan-based composites obtained using agricultural waste [24]. However, the literature studies reveal that so far, no considerable effort has been made to study the removal of CR dye by chitosan-based composites containing the fruit peel of mandarin. In this research, the efficiency of the cross-linked chitosan/*Citrus reticulata* peel waste (C/CRPW) composites in the adsorption of CR dye from an aqueous solution has been investigated. 

Batch experiments were carried out to study the effect of several experimental parameters, such as contact time, pH, adsorbent dosage, initial CR concentration, and temperature. Langmuir, Freundlich, Temkin and Dubinin–Radushkevich isotherm models were used to analyze mechanisms of adsorption. The adsorption kinetics were analyzed by Lagergren’s pseudo-first-order, pseudo-second-order, and Weber–Morris intraparticle diffusion kinetic models. Furthermore, the thermodynamic parameters, such as enthalpy, entropy, and free energy, were also investigated. This study explores the possibility of utilizing CRPW to synthesize cross-linked chitosan-based composites as cost-effective and efficient adsorbents for the adsorptive removal of CR dye from polluted wastewater.

## 2. Materials and Methods

### 2.1. Materials

Chitosan (medium molecular weight, Mw: 190,000–310,000 Da, viscosity 200–800 cP, 1 wt.% in 1% acetic acid, Deacetylation 75–85%) and CR (3,3′-[(1,1′-Biphenyl)-4,4′-diylbis(azo)] bis(4-amino-1-naphthalenesulfonic Acid) 2Na, dye content 97%, CAS 573-58-0) were obtained from Sigma-Aldrich (Sigma-Aldrich Inc., St. Louis, MO, USA). Glutaraldehyde (50 wt.% solution in ethanol) was supplied from Acros Organics (Geel, Belgium). Hydrochloric acid (HCl, 37%) and sodium hydroxide (NaOH, reagent grade) were purchased from JT Baker (Phillipsburg, NJ, USA). All materials reached analytical grade and were used directly.

The CRPW used in this study was obtained from the Seferihisar region of Izmir, Turkey. The CRPW was washed with distilled water and cut into 1 cm^2^ pieces before being dried in an air oven (Natural Convection Oven, JSON-100, JSR, Gongju-City, Chungchungnam-Do, Republic of Korea) at 100 °C. After drying, it was ground into a fine powder and then passed through a sieve to be in the size range of 500 μm to 250 μm.

### 2.2. Preparation of the Cross-Linked C/CRPW Composites

The synthesis processes of cross-linked C/CRPW composites were accomplished based on three main steps. First, the determined amounts of chitosan (1.75 g, 1.50 g, and 1.25 g) were dissolved in 75 mL of acetic acid solutions (5%, *w*/*w*), forming gels, and then mixed with the different amount of CRPW (0.25 g, 0.50 g, and 0.75 g) under stirring with a magnetic stirrer (ISOLAB Laborgeräte GmbH, Eschau, Germany) for 24 h. This was followed by treating C/CRPW powder mixtures with ultrasonic irradiation at 20 kHz, 50% amplitude, and a no pulsation ultrasound regime at 20 min in an ultrasonicator device (Bandelin, HD4100, Berlin, Germany) to accelerate the intercalation of chitosan between CRPW. The mixtures were then added into the NaOH solutions (1.0 M) dropwise to allow the formation of gel beads, and the mixtures were stirred at 150 rpm for another 24 h. After this process, the obtained C/CRPW composites containing different amounts of chitosan and CRWP were filtered and washed multiple times with deionized water until the pH reached 7. In the third stage, the obtained composites were crosslinked with glutaraldehyde solution (2.5%, *v*/*v*) in a water bath at 60 °C for 24 h to enhance their mechanical properties and stability in acidic solutions. Lastly, dried C/CRPW composites were obtained by washing these composites exhaustively with deionized water three times followed by drying in an oven (Natural Convection Oven, JSON-100, JSR, Gongju-City, Chungchungnam-Do, Republic of Korea) at 60 °C for 48 h.

The cross-linked C/CRPW composites containing different amounts of chitosan and CRPW were referred to as xC/yCRPW, where x and y denote the amount of chitosan and CRPW used in the synthesis process, respectively. Three different composites obtained in this study were named 1.75C/0.25CRPW, 1.50C/0.50CRPW, and 1.25C/0.75CRPW.

### 2.3. Characterization of the CRPW and Cross-Linked C/CRPW Composites

Surface morphology and elemental analysis of the cross-linked C/CRPW composites and CRPW were observed with field emission scanning electron microscope (FESEM, Gemini Supra 40 VP, Carl Zeiss, Jena, Germany) and energy dispersive X-ray spectroscopy (EDS), respectively. The samples were coated with a thin layer of gold using a sputter coater (Quorum Q150R ES, Quorum Technologies Ltd., Laughton, UK). The chemical structure of the cross-linked C/CRPW composites was determined using a Fourier transform infrared (FTIR) spectroscope (Thermo Scientific Nicolet iS50FT-IR, Waltham, MA, USA). The spectral data of all composites were acquired in the wavenumber range of 400–4000 cm^−1^ with 50 scans at a resolution of 4 cm^−1^. N_2_ adsorption–desorption was measured at a liquid nitrogen temperature of 77 K using a Quantachrome Autosorb-1C-MS instrument. The specific surface area was determined using the multipoint Brunauer–Emmett–Teller (BET) technique. The pore volume and pore size were derived from the desorption branches of the isotherms using the Barrett–Joyner–Halenda (BJH) method.

Zeta potentials of the CRPW and cross-linked C/CRPW composites were measured over the pH range of 4.0–9.0 on a Zetasizer (Malvern, Zetasizer Nano ZSP, Malvern Panalytical Ltd., Malvern, UK). The zero potential point (pH_pzc_) of composite aerogel was obtained using a zeta potential analyzer.

X-ray diffraction (XRD) measurements were carried out on an X-ray diffractometer (Rigaku-SmartLab, Tokyo, Japan) with Cu Kα radiation at 40 kV and 30 mA in the range of 10–80° at room temperature.

### 2.4. Batch Adsorption Studies

The adsorption of CR dye onto the C/CRPW composites (1.75C/0.25CRPW, 1.50C/0.50CRPW, and 1.25C/0.75CRPW) and CRPW were carried out in batch mode. A stock solution of CR dye (1000 mg/L) was prepared, which was diluted to desired concentrations for further usage. All the adsorption experiments were performed by adding the determined amount of adsorbent to 100 mL CR dye solutions in a thermostatic shaker bath (Nuve ST 30) at a fixed agitation speed of 150 rpm for the pre-determined time. The pH of CR dye solutions was adjusted using HCl (0.1 N) and NaOH (0.1 N) solutions. After adsorption, the concentration of the CR dye was measured spectrophotometrically by monitoring the absorbance at 497 nm using a Hitachi U-2900 spectrophotometer (Hitachi High Technologies Corporation, Tokyo, Japan). 

The equilibrium adsorption capacities of the C/CRPW composites and CRPW (*q_e_* (mg/g)) were estimated using Equation (1):

(1) $$q_{e}=\frac{C_{i}-C_{e}}{m}\times V$$

where *C_i_* is the initial dye concentration (mg/L), *C_e_* is the dye concentration at equilibrium (mg/L), *V* is the volume of the dye solution (L), and m is the mass of the adsorbents (g).

The effects of contact time (0–24 h), initial solution pH (4–9), adsorbent dosage (2–6 g/L), initial CR concentration (20–100 mg/L), and temperature (298.15–328.15 K) on the adsorption of CR dye were investigated. To verify that the results are repeatable, all adsorption experiments were conducted in triplicates and the mean value was reported.

## 3. Results

### 3.1. Characterization of the Cross-Linked C/CRPW Composites

**Figure 1:**
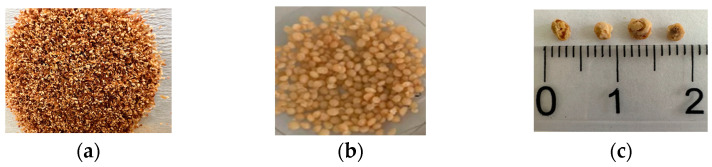
Photographs of fine CRPW (**a**), undried C/CRPW composites (**b**), and dried C/CRPW composites (**c**).

In the synthesis process, spherical composite beads produced by injecting chitosan solution, including CRPW, whose sizes ranged from 500 μm to 250 μm (Figure 1a), into an alkaline bath possess uniform bead sizes 4 mm in diameter. Photographs of the undried and dried C/CRPW composites are shown in Figure 1b and Figure 1c, respectively. The size of the dried C/CRPW composites changes in the range of 2–4 mm (Figure 1c). Although the CRPW, as powder-type adsorbents, have a relatively high adsorption capacity due to their large surface area, they are not preferred for industrial adsorption processes because of being lost easily and damaging the adsorption column. The cross-linked C/CRPW composites, as millimeter-sized spherical adsorbents, are more suitable in the practical adsorption processes due to easy separating and recycling.

**Figure 2:**
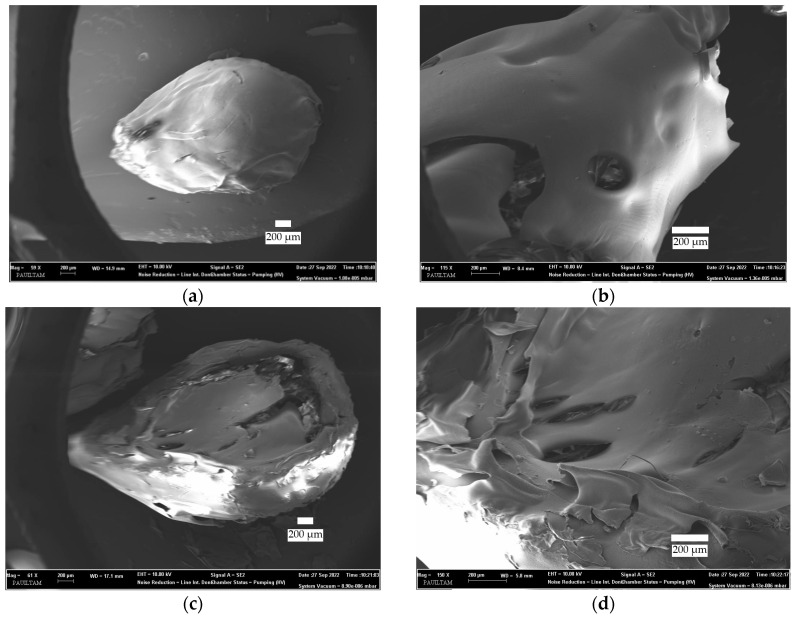

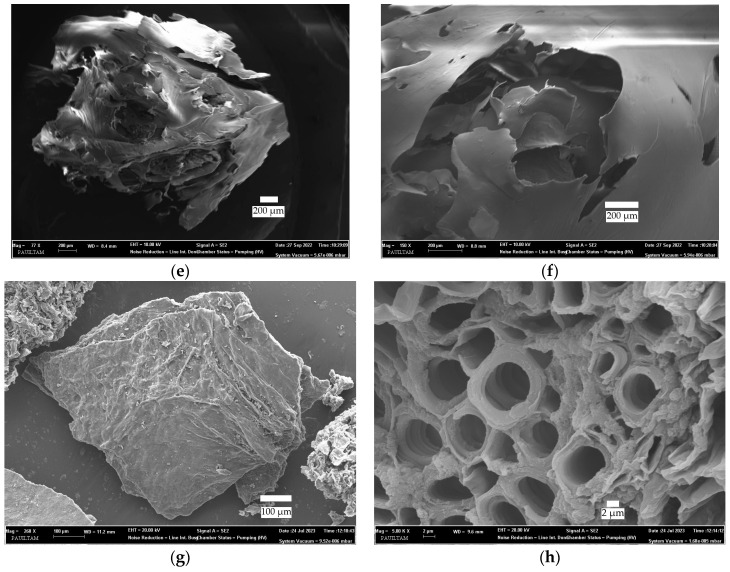
FESEM images of the cross-linked 1.75C/0.25CRPW (**a**,**b**), 1.50C/0.50CRPW (**c**,**d**), and 1.25C/0.75CRPW composites (**e**,**f**) and CRPW (**g**,**h**).

The FESEM images of 1.75C/0.25CRPW, 1.50C/0.50CRPW, and 1.25C/0.75CRPW composites and CRPW are presented in Figure 2. As shown in Figure 2, all the composites have broadly large pores, while many pores of different sizes are observed. The 1.25C/0.75CRPW composites have rougher surfaces, are more porous with well-developed pore openings, and have more irregular shapes than the other composites. The surface roughness of the adsorbents affects the adsorption capacity because it causes high hydrophilicity [25]. SEM image of the CRPW also revealed that there are a great number of holes on the surface of the CRPW.

**Figure 3:**
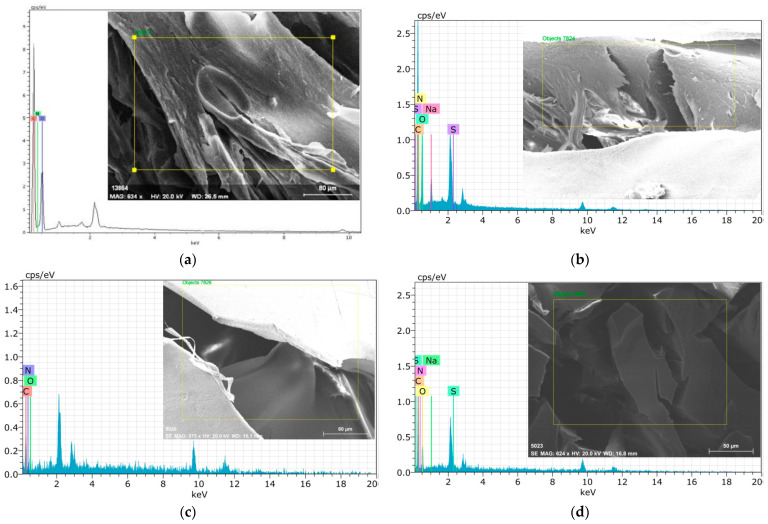

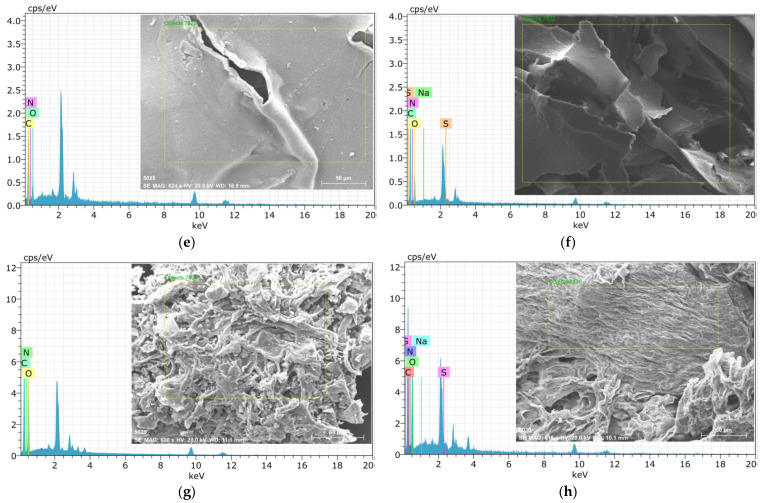
FESEM images and EDS spectra of the cross-linked 1.75C/0.25CRPW (**a**,**b**), 1.50C/0.50CRPW (**c**,**d**), and 1.25C/0.75CRPW composites (**e**,**f**) and CRPW (**g**,**h**) before and after adsorption of CR dye.

Figure 3 shows the FESEM images and EDX spectra of the cross-linked C/CRPW composites and CRPW before and after adsorption of CR dye. As shown in Figure 3, while the cross-linked C/CRPW composites before adsorption show a porous and plate-like surface, they exhibit an agglomerate-like surface after adsorption because the pores are filled with CR molecules. In the EDS spectra of all the cross-linked C/CRPW and CRPW, nitrogen, oxygen, and carbon, which are the main elements of chitosan and CRPW, were observed. In addition, there is an additional sulfur peak in the EDS spectra of cross-linked C/CRPW and CRPW after adsorption of CR dye, which confirms the adsorption of CR dye on cross-linked C/CRPW and CRPW.

**Figure 4:**
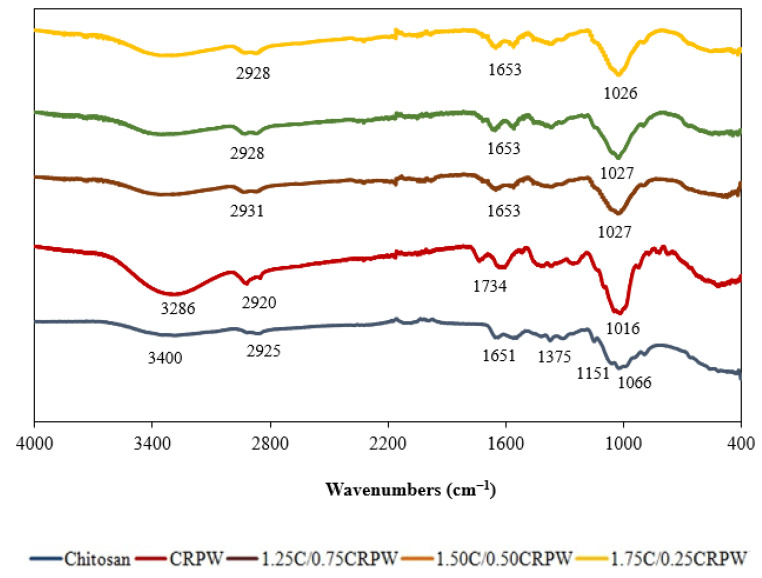
FTIR spectra of chitosan, CRPW, and the cross-linked C/CRPW composites.

The FTIR spectra of chitosan, CRPW, and the cross-linked C/CRPW composites are given in Figure 4. The FTIR spectrum of CRPW showed a broad and intense band at 3286 cm^−1^, corresponding to the O–H stretching vibration that existed in the inter- and intramolecular hydrogen bonding [26]. The bands at 2920 and 1734 cm^−1^ can be attributed to the C–H stretching and C=O stretching vibrations of carboxy groups appearing in CRPW constituents, such as pectin, lignin, and cellulose [27]. The C-H group band at 2920 cm^−1^ of CRPW was moved to around 2930 cm^−1^ in the cross-linked C/CRPW composites, while the C=O group observed at 1734 cm^−1^ of CRPW was moved to 1653 cm^−1^ in the cross-linked C/CRPW composites. The decreased intensity and shifting of these peaks could be a result of lower cellulose content in the cross-linked C/CRPW composites. Furthermore, the strong band at 1016 cm^–1^ represents the C–O–H functional group in CRPW, while it is observed in the cross-linked C/CRPW composites at around 1027 cm^–1^ [16].

The FTIR spectrum of chitosan showed main peaks at 3400, 2925, 1651, 1375, 1151, and 1066 cm^−1^, representing the N–H and O–H stretching vibration, CH_3_ symmetric stretch, C=O stretching vibration, CH_3_ bending vibration, C–O–C bending vibration, and C–OH stretching vibration, respectively. The peaks observed at 1375 cm^−1^ and 1151 cm^−1^ in the FTIR spectrum of chitosan disappeared in the FTIR spectrum of the cross-linked C/CRPW composites because of the cross-linking of chitosan with glutaraldehyde. Furthermore, the spectrum of the cross-linked C/CRPW composites show a new peak at 1650 cm^−1^ that can be attributed to amide (-C(=O)N-) due to the cross-linking reaction of chitosan and glutaraldehyde [28].

**Figure 5:**
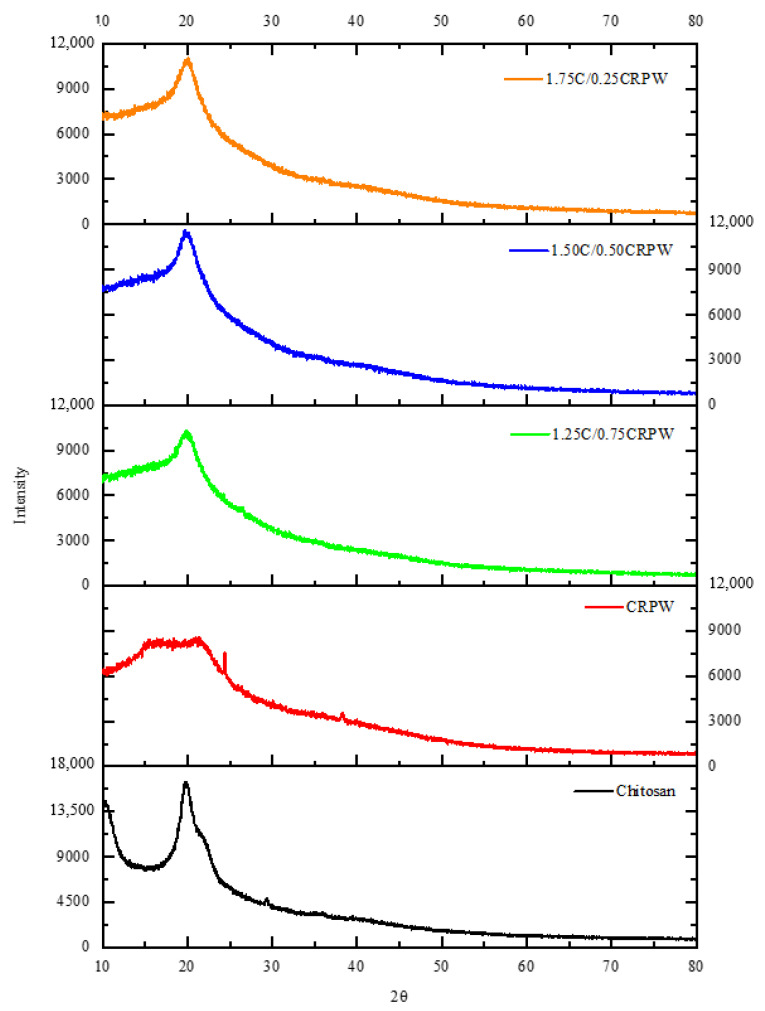
XRD patterns for chitosan, CRPW, and the cross-linked C/CRPW composites.

The XRD patterns of the chitosan, CRPW, and cross-linked C/CRPW composites are shown in Figure 5. In the XRD pattern of the CRPW, broad peaks around 2θ = 15–21° indicate the amorphous structure of CRPW due to the presence of a large amount of pectin. In addition, sharp peaks at 2θ = 24.4° and other small sharp peaks originated from inorganic substances [29]. In the XRD patterns of the C/CRPW composites, while the peak at around 2θ = 25° is not evident for 1.75C/0.25CRPW and 1.50C/0.50CRPW composites, it becomes evident for the 1.75C/0.25CRPW composites due to the increasing ratio of CRPW in the composite structure. The XRD pattern of the chitosan has characteristic crystalline diffraction peaks at 2θ = 19.9° and 2θ = 29.4° [30]. The small peak at 2θ = 29.4° is not observed in the patterns of the C/CRPW composites. Additionally, the diffraction peak at 2θ = 19.9° broadened and its intensity decreased. The reason is that the incorporation of CRPW in the chitosan-based composite causes a decrease in crystallinity due to the disordered intercalated structure.

**Figure 6:**
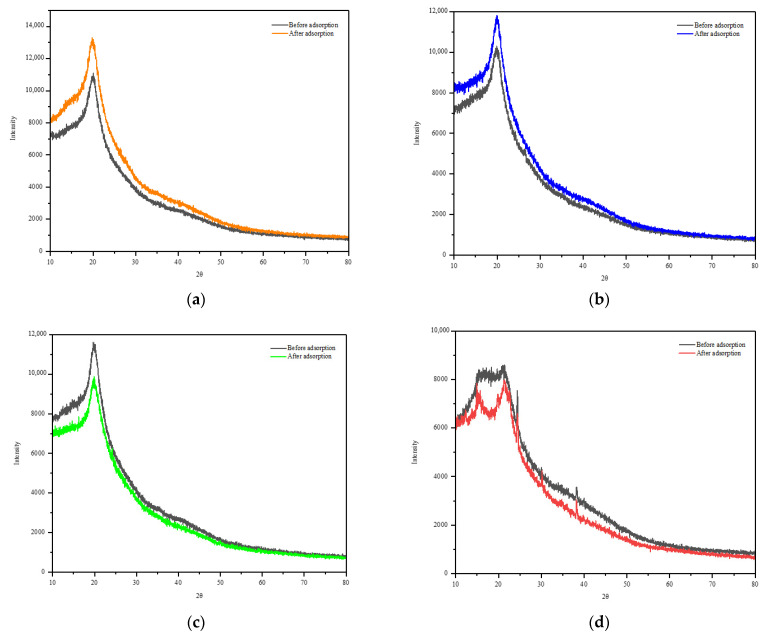
XRD patterns of the 1.75C/0.25CRPW (**a**), 1.50C/0.50CRPW (**b**), and 1.25C/0.75CRPW composites (**c**) and CRPW (**d**) before and after adsorption of CR dye.

The diffraction pattern of the C/CRPW composites did not significantly change after CR dye adsorption (Figure 6), indicating that the crystallinity of the C/CRPW composites was not changed by the adsorption process. However, it is evident from the XRD results that significant differences occurred in the peak intensities for the C/CRPW composites before and after adsorption. As shown in Figure 6, a more significant change was observed between the diffraction patterns of CRPW before and after adsorption. The change could be due to the hydrogen bonds formed during the adsorption of CR onto the surface of CRPW [31].

A nitrogen isothermal adsorption technique was employed to investigate the textural properties and pore structure of the CRPW and cross-linked C/CRPW composites. BET specific surface area, average pore diameter, and total pore volume based on the BJH theory of the sample were measured and are represented in Table 1. It has been found that the increase of the CRPW amount in the chitosan matrix leads to an increase in the surface area and total pore volume of the C/CRPW composites. This result explains that the interfacial interaction between the chitosan matrix and CRPW greatly affects the material pore structure [32].

**Figure 7:**
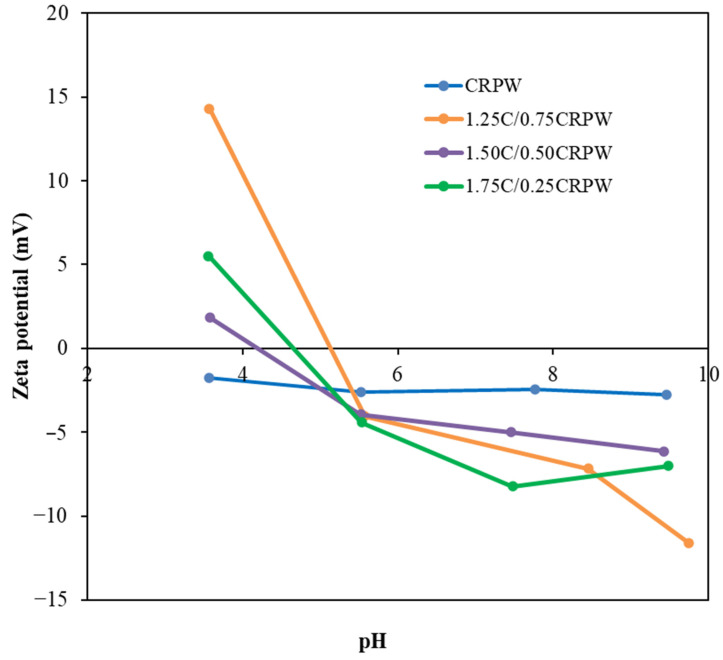
pH-dependent zeta potentials of the CRPW and cross-linked C/CRPW composites.

The effect of solution pH on the surface of CRPW and crosslinked C/CRPW composites was examined by zeta potential measurements and the results are shown in Figure 7. The zero-point charge (pH_zpc_) values were found to be 5.13, 4.20, and 4.65 for the cross-linked 1.25C/0.75CRPW, 1.50C/0.50CRPW, and 1.75C/0.25CRPW composites, respectively. At a pH of 4, the higher zeta potential value of 1.25C/0.75CRPW composites compared to the other adsorbents shows that the cross-linked 1.75C/0.25CRPW composites are more favorable to adsorb CR anions [33]. This result also supports the batch adsorption results obtained in this study.

### 3.2. Effect of Contact Time and Adsorption Kinetic Models

**Figure 8:**
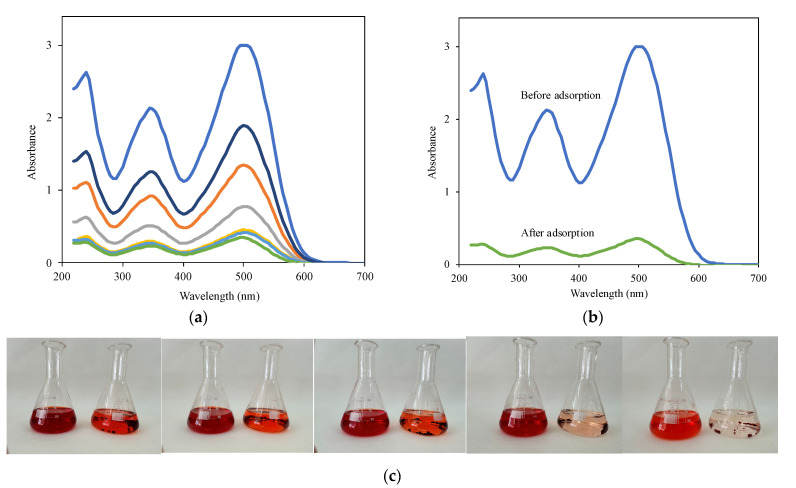
Removal of CR dye from aqueous solution by the cross-linked C/CRPW composites. (**a**) Time-dependent absorption spectra of 60 mg/L of CR in the presence of 1.25C/0.75CRPW (2.0 g/L). (**b**) Absorption spectra of 60 mg/L of CR before and after addition of 1.25C/0.75CRPW (2.0 g/L). (**c**) Photographs of 100, 80, 60, 40, and 20 mg/L of CR solutions before and after addition of the cross-linked 1.25C/0.75CRPW composites.

Anionic CR was chosen to examine the adsorption capacity of the CRPW and the cross-linked C/CRPW composites. In adsorption studies, an increase in the contact time of the prepared adsorbents with CR led to a progressive reduction in their absorption intensity (Figure 8a) and a remarkable reduction in the absorption intensity of CR after adsorption (Figure 8b). In addition, a visible color change in CR solutions was observed during the adsorption process (Figure 8c).

**Figure 9:**
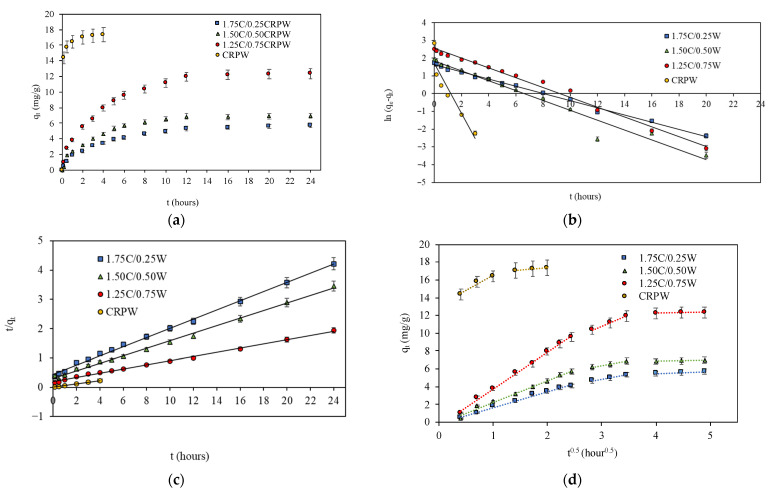
Adsorption amount of CR onto the cross-linked C/CRPW composites and CRPW varying with time (**a**) and linear fitting of Lagergren’s pseudo-first-order (**b**), pseudo-second-order (**c**), and intraparticle diffusion (**d**). (Experimental conditions: adsorbent dosage = 2 g/L; initial dye concentration = 60 mg/L; pH = 7; temperature = 298 K).

The effect of contact time on the adsorption capacities of the cross-linked C/CRPW composites and CRPW is shown in Figure 9a. As shown in Figure 9a, the adsorption capacities of the C/CRPW composites increase with contact time and reach equilibrium after 24 h. However, the increase in adsorption capacity values is relatively higher during the initial 12 h due to plenty of vacant sites available for the adsorption of CR molecules. Consequently, a large mass transfer rate was observed during the initial 12 h of the process, and equilibrium was achieved at approximately 95% efficiency. After that, a gradual decrease in the adsorption rate is observed until 24 h as the empty spaces on the adsorbent surface gradually decrease. After 24 h, equilibrium was achieved for all the cross-linked C/CRPW composites. 

When the effect of contact time on the adsorption capacity of CRPW was examined, it was observed that the adsorption of CR dye on CRPW was much faster than that of the C/CRPW composites. During the initial 1 h of the adsorption process of CRPW, equilibrium was achieved at approximately 95% efficiency, and the equilibrium was achieved after 4 h. In addition, it has been observed that CRPW has a much higher adsorption capacity than those of the C/CRPW composites at equilibrium.

Lagergren’s pseudo-first-order and pseudo-second-order kinetic models were used to evaluate the adsorption kinetics of CR onto the cross-linked C/CRPW composites and CRPW.

Lagergren’s pseudo-first-order kinetic model [34]:

(2) $$\ln\left(q_{e}-q_{t}\right)=\ln q_{e}-k_{1}\cdot t$$

Pseudo-second-order kinetic model [35]:

(3) $$\frac{t}{q_{t}}=\frac{1}{k_{2}\cdot q_{e}^{2}}+\frac{t}{q_{e}}$$

where *q_e_* and *q_t_* (mg/g) are the adsorption amount of CR at equilibrium and *t* (hour), respectively; *k*_1_ (1/hour) and *k*_2_ (g/mg hour) are the rate constants of the Lagergren’s pseudo-first-order and pseudo-second-order model, respectively.

The representative graphs for Lagergren’s pseudo-first-order and pseudo-second-order equations are displayed in Figure 9b,c, respectively, and the kinetic model parameters are shown in Table 2.

It can be seen from the data in Table 2 that the pseudo-second-order model has a higher correlation coefficient (*R*^2^ > 0.99) for CR adsorption onto all the cross-linked C/CRPW composites and CRPW. The adsorption capacities of 1.75C/0.25CRPW, 1.50C/0.50CRPW, and 1.25C/0.75CRPW composites and CRPW were 5.69, 6.94, 12.35, and 17.35 mg/g, respectively. The data also showed that the adsorption capacity of the C/CRPW composites increases with increasing CRPW contents. The 1.25C/0.75CRPW composites exhibited the best adsorption performance among the composites. This result is due to the addition of CRPW increasing the porosity and surface area of the C/CRPW composites, as well as the high adsorption capacity of CRPW for the CR dye molecules. This result is supported by the SEM images shown in Figure 2.

The Weber–Morris intraparticle diffusion model was also studied to better understand the adsorption mechanism of CR dye onto the cross-linked C/CRPW composites and CRPW.

Equation (4) shows the linear form of the intraparticle diffusion kinetic model [36]:

(4) $$q_{t}=C+K_{id}\cdot t^{0.5}$$

where *q_t_* is the adsorption amount of CR at *t* (hour), *t* is adsorption time (hour), *K_id_* (mg/g hour^0.5^) is intraparticle diffusion rate constant, and *C* is mass transfer residence due to the thickness of the boundary layer.

The intraparticle diffusion plots of adsorption capacity *q_t_* versus *t*^0.5^ for the adsorption of CR onto the cross-linked C/CRPW composites and CRPW are shown in Figure 3d. The kinetic parameters, *K_id_* and *C*, were determined by the intercept and slope of linear plots and are given in Table 2. As can be seen from Figure 9d, the linear lines at early contact times did not pass through the origin, suggesting that there was a boundary layer effect and internal diffusion was not merely a rate-controlling step. In other words, the adsorption of the CR molecules onto the cross-linked C/CRPW composites and CRPW was controlled by more than one adsorption rate process. The phenomena can be explained by the presence of micropores in the cross-linked C/CRPW composites and CRPW [37].

Three distinct stages for the adsorption of CR onto the cross-linked 0.25W/1.75CRPW, 0.50W/1.50CRPW, and 0.75W/1.25CRPW composites were identified from the fitting of Equation (4). The stages were 0–6 h, 8–12 h, and 16–24 h, representing the first, second, and third stages, respectively. During the first stage, at the beginning of the process, the adsorption speed was faster and the diffusion of CR molecules from the solution to the external surface of cross-linked C/CRPW composites played an essential role, which was related to external diffusion. In the second stage, a slower adsorption rate was observed, referring to intraparticle diffusion (internal diffusion). Subsequently, in the third stage, which acquired a kinetic balance, the equilibrium of adsorption and desorption was observed [19]. In addition, as shown in Table 2, the diffusion rate constant values in the first and second stages for 1.25C/0.75CRPW composites were higher than those for 1.50C/0.50CRPW and 1.75C/0.25CRPW composites. The higher the *K_id_* value, the easier the diffusion and transport into the pores of adsorbents are [38]. According to these results, the increasing amount of CRPW in the composition of composites causes the increasing diffusion and transportation of CR molecules into the interior of the composites.

### 3.3. Effect of Initial Solution pH

**Figure 10:**
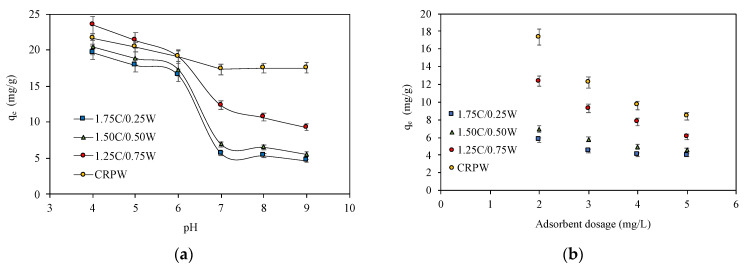

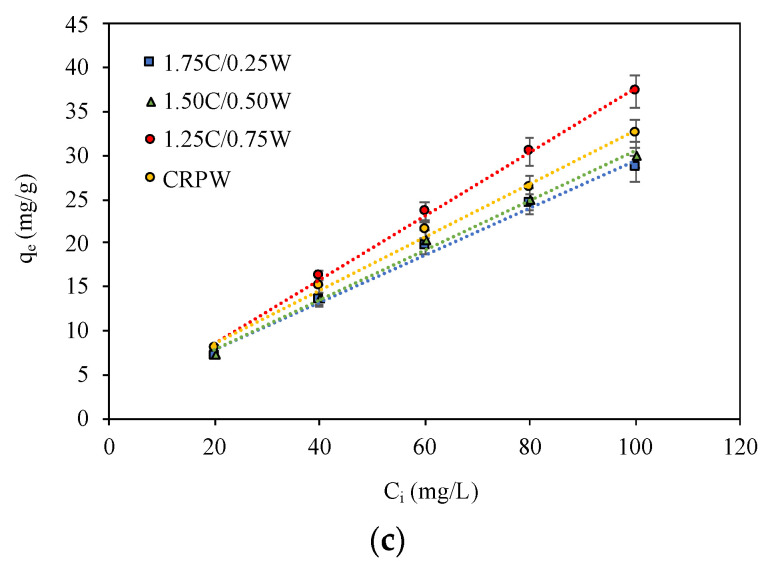
Effect of initial solution pH (**a**), adsorbent dosage (**b**), and initial dye concentration (**c**) on the adsorption of CR onto the cross-linked 1.75C/0.25CRPW, 1.50C/0.50CRPW, and 1.25C/0.75CRPW composites and CRPW. (Experimental conditions: adsorbent dosage = 2 g/L; contact time = 24 h; initial dye concentration = 60 mg/L; temperature = 298 K).

The initial solution pH is a significant parameter in adsorption processes because it influences the chemistry of both the adsorbate and the adsorbents. CR is a pH-sensitive dye, resulting in the dye changing color from red to blue. The original pH of the CR solution was around 7.0. The adsorption of CR on the cross-linked C/CRPW composites was studied in the pH range of 4–9, in which range the color of CR is stable and red. Figure 10a shows the effect of initial solution pH on the adsorption of CR by the C/CRPW composites and CRPW. As shown in Figure 10a, there was a decrease in the adsorptive capacity of all the composites with an increasing initial solution pH, indicating that the adsorption of CR by the composites was pH dependent. The highest adsorptive capacity for all the composites (1.75C/0.25CRPW 19.63 mg/g; 1.50C/0.50CRPW 20.41 mg/g; 1.25C/0.75CRPW 23.48 mg/g) was obtained at the lowest pH of 4. The reason for this result is that the surface charge of the cross-linked C/CRPW composites becomes the highest value at pH 4. At lower pH values than pH_PZC_, zeta potential is positive and indicates that the surface charge of the adsorbents is positive, and they easily attract anionic CR dye molecules. The electrostatic attraction between protonated C/CRPW composites and the anionic CR dye enhances the adsorption capacity of the C/CRPW composites in low pH values. In this study, the zeta potential value of CRPW is negative in the pH range of 4 and 9, which were the pH values that the adsorption studies were carried out with. However, the adsorption property of CRPW in this pH range is also effective, which indicates that there are other mechanisms involved besides the electrostatic attraction taking place in the adsorption process [39].

### 3.4. Effect of Adsorbent Dosage

Adsorbent dosage is a crucial factor in the adsorption process due to impacts on both the process cost and the removal percentage of contaminants. The effect of the adsorbent dosage on the CR adsorption on the C/CRPW composites and CRPW was examined by increasing the adsorbent dosage from 2 to 6 g/L at constant experimental conditions, and the results are shown in Figure 10b. By increasing the adsorbent dosage, more adsorption sites are available for CR dye molecules, resulting in the reduction of the adsorption capacity of adsorbents. As seen in Figure 10b, as the adsorbent dosage increased from 2 to 6 g/L, the adsorption capacity decreased from 5.69 to 3.98 mg/g, 6.94 to 4.58 mg/g, 12.35 to 6.07 mg/g, and 17.35 to 8.40 mg/g for the 1.75C/0.25CRPW, 1.50C/0.50CRPW, and 1.25C/0.75CRPW composites and CRPW, respectively. A total of 2 g/L of adsorbent dosage was selected as an optimum dosage for further experiments due to more active sites of the composites remaining unsaturated during the CR dye adsorption process.

### 3.5. Effect of Initial CR Concentration

The effect of the initial CR dye concentration on the adsorption capacities of the cross-linked C/CRPW composites and CRPW was investigated by changing the dye concentration from 20 to 100 mg/L at constant experimental conditions. As shown in Figure 10c, when the initial dye concentration was increased to 20 and 100 mg/L, the adsorption capacities of 1.75C/0.25CRPW, 1.50C/0.50CRPW, and 1.25C/0.75CRPW composites and CRPW increased from 7.10 to 28.52 mg/g, 7.31 to 30.00 mg/g, 8.06 to 37.21 mg/g, and 7.86 to 32.42 mg/g, respectively. The adsorption capacities of all the composites were improved by increasing the CR dye concentration in the aqueous solution due to the higher collision probability between the active sites of the composites and the CR dye molecules [40].

### 3.6. Adsorption Isotherms

The nature of the adsorption process of CR onto the cross-linked C/CRPW composites and CRPW was examined with Langmuir [41], Freundlich [42,43], Temkin [44], and Dubinin–Radushkevich [45] isotherm models. The linear forms of these isotherm models can be expressed as follows:

Langmuir:

(5) $$\frac{C_{e}}{q_{e}}=\frac{C_{e}}{q_{max,L}}+\frac{1}{K_{L}q_{max,L}}$$

Freundlich:

(6) $$\ln q_{e}=\ln K_{F}+\left(1/n\right)\ln C_{e}$$

Temkin:

(7) $$q_{e}=B_{T}\ln K_{T}+B_{T}\cdot \ln C_{e}$$

Dubinin–Radushkevich:

(8) $$\ln q_{e}=\ln q_{max,D-R}-K_{D-R}\cdot \varepsilon^{2}$$

where *K_L_* is the Langmuir equilibrium constant (L/mg) used to calculate the equilibrium dimensionless parameter (*R_L_*), which is defined by $R_{L}=1/(1+K_{L}\cdot C_{i})$ and determines the feasibility of the Langmuir model; *q_max,L_* is the Langmuir maximum monolayer adsorption capacity (mg/g); *K_F_* is the Freundlich constant, also known as adsorption capacity ((mg/g)(mg/L)^−1/*n*^), which describes the adsorption capacity; 1/*n* is the Freundlich intensity parameter representing surface heterogeneity; *K_T_* is the Temkin isotherm equilibrium binding constant (L/mg); *B_T_* is the Temkin constant, i.e., heat of adsorption (J/mol); *q_max,D_*_–*R*_ is the Dubinin–Radushkevich maximum adsorption capacity (mg/g); *K_D-R_* is a constant related to sorption energy (mol^2^/kJ^2^); and *ε* is the Polanyi potential (kJ/mol).

The ε value can be calculated using Equation (9):

(9) $$\varepsilon =R\cdot T\cdot \ln\left(\frac{C_{e}+1}{C_{e}}\right)$$

where *R* of 8.314 J/mol·K is the universal gas constant and *T* is the absolute temperature in Kelvin.

The mean free energy of adsorption (*E*, kJ/mol) can be calculated using Equation (10):

(10) $$E=\frac{1}{\sqrt{2K_{D-R}}}$$

Free energy can determine the type of adsorption process. If *E* is less than 8 kJ/mol, the adsorption process is physisorption, while if it is more than 8 kJ/mol, the process is chemisorption.

**Figure 11:**
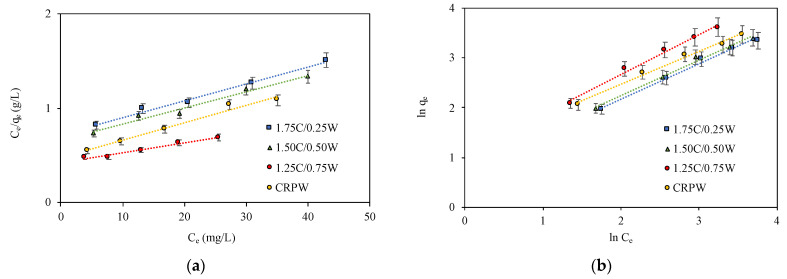

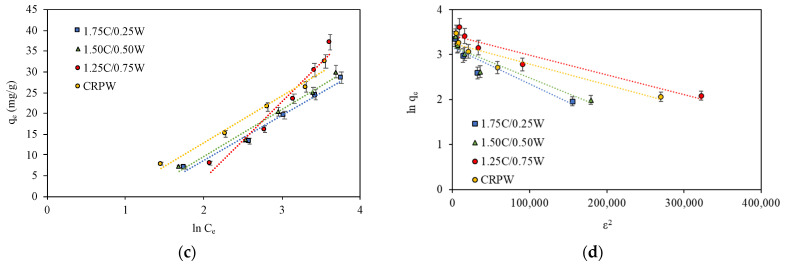
Langmuir (**a**), Freundlich (**b**), Temkin (**c**), and Dubinin–Radushkevich (**d**) adsorption isotherm models of CR on the cross-linked 1.75C/0.25CRPW, 1.50C/0.50CRPW, and 1.25C/0.75CRPW composites and CRPW. (Experimental conditions: adsorbent dosage = 2 g/L; contact time = 24 h; pH = 4; temperature = 298 K).

The linear fitting for the Langmuir, Freundlich, Temkin, and Dubinin–Radushkevich isotherms are shown in Figure 11, and the calculated isotherm parameters are listed in Table 3.

The values of correlation coefficient *R*^2^ were used to determine suitable isotherms, which explain the adsorption process. In this study, the Freundlich isotherm model was best fitted for all the cross-linked C/CRPW composites and CRPW with *R*^2^ values of 0.9889–0.9908. Hence, it can thus be concluded that the adsorption process takes place on a heterogeneous surface of the C/CRPW composites and CRPW. Among the adsorbents, CRPW has the highest *K_F_* value. When the *K_F_* values of the cross-linked C/CRPW composites are compared, it was observed that *K_F_* values increased from 2.115–2.886 as the amount of CRPW in the composite contents increased from 0.25 g to 0.75 g, which confirmed the above kinetic and equilibrium studies where dye adsorption capacity increased as CRPW contents in the composites were raised.

The maximum adsorption capacities of the 1.75C/0.25CRPW, 1.50C/0.50CRPW, and 1.25C/0.75CRPW composites and CRPW were calculated using the Langmuir isotherm equation (Equation (5)) and were found to adsorb CR at 55.56 mg/g, 58.48 mg/g, 97.09 mg/g, and 54.05 mg/g, respectively. Although the *R*^2^ values of the Langmuir isotherm model (*R*^2^ = 0.9714–0.9895) were low, the calculated *R_L_* values (0.2034–0.6729), as shown in Table 3, were found to be between zero and one, indicating that the adsorption was a favorable process.

The heat of adsorption (*B_T_*) of all the composites calculated from the Temkin isotherm model was found to increase from 10.88 to 18.56 (J/mol) as the amount of CRPW was raised in the structure of the cross-linked C/CRPW composite. The relatively high correlation coefficient values (*R*^2^ = 0.9520–0.9844) of the Temkin model for all cross-linked C/CRPW composites and CRPW showed the suitability of the model in interpreting the adsorption process. The values of *E* (0.250–0.353 kJ/mol), calculated from the Dubinin–Radushkevich isotherm model using Equation (10), were found to be <8 kJ/mol, indicating that physisorption plays a dominant role in the adsorption of CR dye onto all the cross-linked C/CRPW composites and CRPW [46]. The low correlation coefficient (*R*^2^ = 0.8631–0.9049) of the Dubinin–Radushkevich model indicated the weakness of the model in clarifying the adsorption process.

### 3.7. Effect of Temperature and Adsorption Thermodynamics

Thermodynamic studies for the adsorption of CR dye on the 1.75C/0.25CRPW, 1.50C/0.50CRPW, and 1.25C/0.75CRPW composites and CRPW were carried out within temperature-dependent adsorption in the range of 298–328 K. The temperature effect on the adsorption capacities of all the composites and CRPW for CR dye indicated that the uptake capacity of CR dye increases while increasing the temperature from 298 K to 328 K. The result shows that the adsorption of CR dye onto the cross-linked C/CRPW composites and CRPW is an endothermic reaction. The temperature of the conventional textile industry wastewater is about 308–318 K [47]. However, the temperature of textile wastewater varies considerably and may reach 343 K at the outlet of dye processes [48]. In this study, 318 K was chosen as the adsorption temperature, at which the temperature of C/CRPW composites exhibit maximum adsorption capacities due to their endothermic nature. Although the adsorption capacity increases with a rise in temperature, higher temperatures are not preferred considering the discharge limit temperature of textile effluent (e.g., 303 K for the Canadian Council of Ministers of the Environment (CCME), 323 K for the Bureau of Indian Standards) [49].

Thermodynamic parameters, including Gibbs free energy change (Δ*G*°), enthalpy change (Δ*H*°), and entropy change (Δ*S*°) of the adsorption process, were calculated using the following equations [50]:

(11) ∆G°=∆H°−T·∆S°

(12) $$K_{C}=q_{e}/C_{e}$$

(13) $$\ln K_{C}=\frac{∆S{}^{\circ}}{R}-\frac{∆H{}^{\circ}}{R\cdot T}$$

where *R* is the gas constant (8.314 J/mol·K), *T* is the absolute temperature (K), and *K_C_* represents the distribution coefficient.

**Figure 12:**
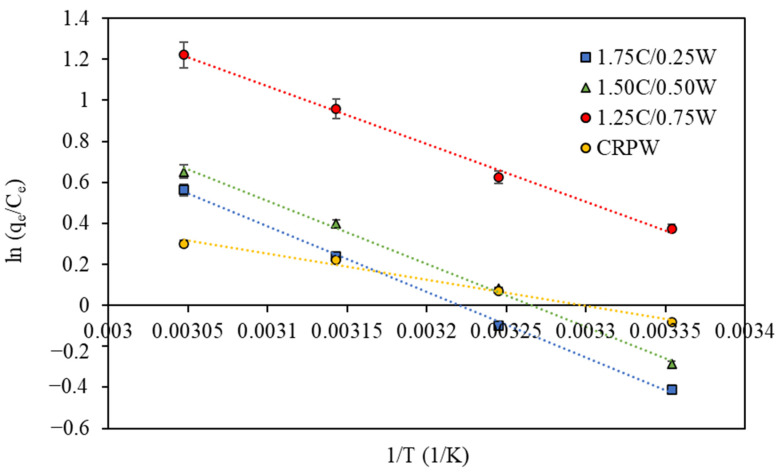
Van’t Hoff plot for the adsorption of CR dye onto the cross-linked 1.75C/0.25CRPW, 1.50C/0.50CRPW, and 1.25C/0.75CRPW composites and CRPW. (Experimental conditions: adsorbent dosage = 2 g/L; contact time = 24 h; initial dye concentration = 100 mg/L; pH = 4).

The values of Δ*H*° and ΔS° can be determined from the van’t Hoff plot, which is the linear plot of In *K_C_* versus 1/*T* (Figure 12) from the slope and intercept, respectively. The thermodynamic parameters are presented in Table 4. 

The negative values of Δ*G*° suggest the spontaneity of the adsorption processes. As shown in Table 4, the higher absolute values of Δ*G*° were observed at higher temperatures for all the cross-linked C/CRPW composites and CRPW. Thus, the adsorption of CR dye onto the cross-linked C/CRPW composites and CRPW is more spontaneous and favorable at higher temperatures. However, at lower temperatures, the positive values of Δ*G*° for the adsorption of CR dye onto the 1.75C/0.25CRPW and 1.50C/0.50CRPW composites and CRPW indicate the nonspontaneous and unfavorable adsorption process. 

The adsorption of CR dye onto the 1.75C/0.25CRPW, 1.50C/0.50CRPW, and 1.25C/0.75CRPW composites and CRPW had enthalpy changes of 26.465, 25.528, 23.358, and 10.572 kJ/mol, respectively. The positive and lower than 40 kJ/mol of Δ*H*° values confirm that it is an endothermic process in physical interaction between CR molecules and all the cross-linked C/CRPW composites and established CRPW. In addition, the positive values of Δ*S*° suggest increased randomness at the solid–solution interface during the adsorption of CR dye onto all the cross-linked C/CRPW composites and CRPW [51].

### 3.8. Reusability Studies

**Figure 13:**
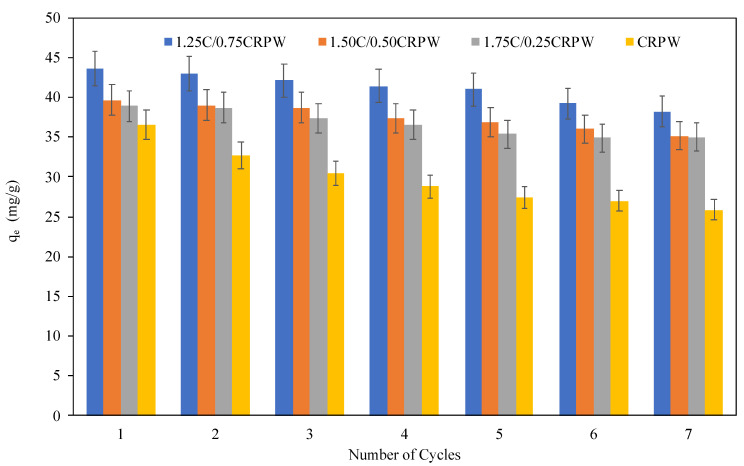
Recycling of the cross-linked 1.75C/0.25CRPW, 1.50C/0.50CRPW, and 1.25C/0.75CRPW composites and CRPW for the removal of CR.

The reusability of 1.75C/0.25CRPW, 1.50C/0.50CRPW, and 1.25C/0.75CRPW composites and CRPW was investigated by six cycles of adsorption–desorption process, and the results are shown in Figure 13. NaOH solution was used in desorption studies to facilitate the diffusion of CR molecules from the active sites of the composites. The recovered composites were washed with deionized water several times and dried for the next adsorption test. As shown in Figure 13, the adsorption capacities of the cross-linked C/CRPW composites and CRPW for CR gradually decreased with the increasing number of cycles. However, after six adsorption–desorption cycles, the 1.75C/0.25CRPW, 1.50C/0.50CRPW, and 1.25C/0.75CRPW composites and CRPW remained 90.0, 88.7, 87.7, and 70.9% of its initial CR adsorption capabilities, respectively. The high *q_e_* values of the cross-linked C/CRPW composites are due to the improved mechanical stability of the composites by the cross-linking of chitosan containing CRPW with glutaraldehyde [24]. The recyclability studies reveal that the cross-linked C/CRPW composites, as bio-adsorbents, show high performance and sustainability in practical applications.

### 3.9. Adsorption Mechanism of CR by the CRPW and Cross-Linked C/CRPW Composites

**Figure 14:**
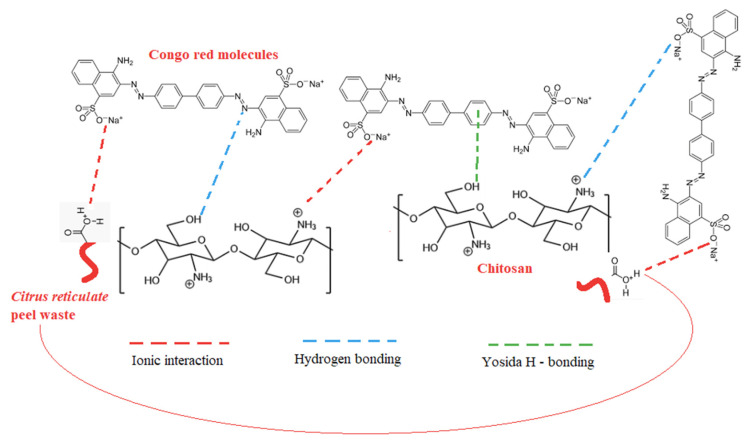
Schematic diagram depicting the interaction of CR molecules with the CRPW and cross-linked C/CRPW composites.

The schematic representation of CR molecules’ possible interaction with the crosslinked C/CRPW composites is shown in Figure 14. It illustrates that the adsorption mechanism of C/CRPW composites with CR dye macromolecules fundamentally depends on the π–π bond, hydrogen bond, and electrostatic interaction. In an aqueous solution, CR dye can dissolve, giving the dye anions (CR-SO_3_^−^). Chitosan has polar functional groups (-NH_2_ and -OH) on its surface. At lower pH values, strong electrostatic interactions between the negatively charged sulfonated groups (-SO_3_^−^) of the CR dye molecules and the positively charged amine group (-NH_3_^+^) of the chitosan enhance the adsorption capacity of the adsorbents. The electrostatic interaction might be the main mechanism for the adsorption of CR on the C/CRPW composites. The other interactions are hydrogen bonding, which occurs between the hydroxyl group of chitosan and electronegative residues in the dye molecule, and the Yoshida H-bonding, which can also occur between the hydroxyl group of chitosan and the aromatic ring in dye [52].

According to the FTIR results of CRPW, -OH, COC and -COOH groups are present on the surface of CRPW. The carboxylic and hydroxylic groups consume H^+^ and are protonated in acidic solutions. Then, the positively charged -OH_2_^+^ and -COOH_2_^+^ molecules in an aqueous solution can exist as cationic hydroxides interact with CR through electrostatic interaction. In addition, CR adsorption by the CRPW can occur by the π–π bond and hydrogen bond due to the presence of heteroatoms comprising oxygen and nitrogen on the surface of CRPW besides electrostatic interaction [29,53].

### 3.10. Comparative Study

The maximum adsorption capacities of 1.75C/0.25CRPW, 1.50C/0.50CRPW, and 1.25C/0.75CRPW composites and CRPW are found to be 38.92 mg/g, 39.66 mg/g, 43.57 mg/g, and 36.50 mg/g, respectively. Table 5 shows the maximum adsorption capacities of different adsorbents obtained by agricultural waste and chitosan-based composites for CR dye. CRPW, which was used as an adsorbent in this study, has a relatively high adsorption capacity compared to other agricultural waste. In this study, the highest adsorption capacity (43.57 mg/g) was obtained for the 1.25C/0.75CRPW composites. The adsorption capacity of C/CRPW composites was enhanced with an increasing CRPW content. As shown in Table 5, some chitosan-based composites reported in the literature show relatively higher adsorption capacities. However, most of them are costly due to the use of commercially valuable polymers in their production. Therefore, the C/CRPW composites are preferred for use as an adsorbent for removing CR due to their excellent properties, such as being readily available, environment-friendly, and cost-effective. 

## 4. Conclusions

In the present research work, three cross-linked chitosan-based composites containing mandarin (*Citrus reticulata* Seferihisar cv.) peel waste, namely 1.75C/0.25CRPW, 1.50C/0.50CRPW, and 1.25C/0.75CRPW, as new low-cost and green adsorbents have been successfully synthesized for the removal of the toxic anionic dye, CR, from aqueous solutions. 1.25C/0.75CRPW composites performed the best adsorption capacity (43.57 mg/g) for CR removal from aqueous solutions, followed by 1.50C/0.50CRPW composites (39.66 mg/g) and 1.75C/0.25CRPW composites (38.92 mg/g). The results showed that the increase in CRPW content of the cross-linked C/CRPW composites increased adsorption capacity because of the enhancement of the porosity of the composite surface. The influence of various operating parameters, such as contact time, pH, adsorbent dosage, initial dye concentration, and temperature on the adsorption capacities of all the composites, was investigated. Compared with the effect of factors on the adsorption capacities of the composites, all parameters influenced the adsorption capacities; however, the effect of pH change on the adsorption capacities was more obvious. The highest adsorption capacity values were obtained in adsorption conditions with a pH of 4, adsorbent dosage of 2 g/L, initial CR dye concentration of 100 mg/L, and adsorption temperature of 328 K. The adsorption kinetics for all the composites investigated in this work followed the pseudo-second-order equation. The adsorption equilibrium data could also be well described by the Freundlich isotherm model. The high adsorption ability of the cross-linked C/CRPW composites and the abundant availability of CRPW as waste material revealed that these composites can be used as low-cost and green bio-adsorbents for the removal of CR dye in industrial wastewater.

## Figures and Tables

**Table 1:** Surface parameters of the CRPW and cross-linked C/CRPW composites.

Samples	BET Surface Area (m^2^/g)	BJH Pore Volume (cm^3^/g)	BJH Pore Size (A°)
1.75C/0.25CRPW	10.04	0.06225	29.83
1.50C/0.50CRPW	13.86	0.1014	16.17
1.25C/0.75CRPW	20.42	0.1500	16.39
CRPW	17.61	0.02269	18.85

**Table 2:** Kinetic parameters of the Lagergren’s pseudo-first-order, pseudo-second-order, and intraparticle diffusion kinetic models for CR onto the cross-linked C/CRPW composites and CRPW.

Models	Parameters	Values			
		1.75C/0.25CRPW	1.50C/0.50CRPW	1.25C/0.75CRPW	CRPW
Experimental result	*q_e,exp_* (mg/g)	5.69	6.94	12.35	17.35
Lagergren’s pseudo-first-order	*q_e,cal_* (mg/g)	5.09	6.34	12.81	5.59
	*k*_1_ (1/h)	0.2021	0.2775	0.2763	1.4133
	*R* ^2^	0.9950	0.9652	0.9893	0.8725
Pseudo-second-order	*q_e,cal_* (mg/g)	6.39	7.78	13.91	17.54
	*k*_2_ (g/mg h)	0.0545	0.0548	0.0275	1.0481
	*R* ^2^	0.9971	0.9963	0.9968	0.9999
Intraparticle diffusion	*k_id,_*_1_ (mg/g h^0.5^)	1.7985	2.4436	4.0972	3.4778
	*C_1_*	−0.1430	−0.2177	−0.3548	13.075
	*R* ^2^	0.9901	0.9876	0.9968	0.9623
	*k_id,_*_2_ (mg/g h^0.5^)	1.0920	1.0936	2.4286	0.5309
	*C*_2_	1.5399	3.0752	3.5203	16.296
	*R* ^2^	0.9954	0.9997	0.9984	0.9851
	kid,3 (mg/g h^0.5^)	0.2411	0.1211	0.1380	-
	*C*_3_	4.5149	6.3582	11.682	-
	*R* ^2^	0.9972	0.9551	0.9870	-

**Table 3:** Adsorption isotherm parameters for adsorption of CR onto the cross-linked C/CRPW composites and CRPW.

Isotherm	Constants	1.75C/0.25CRPW	1.50C/0.50CRPW	1.25C/0.75CRPW	CRPW
Langmuir	*q_max,L_* (mg/g)	55.56	58.48	97.09	54.05
	*K_L_* (L/mg)	0.0251	0.0260	0.0243	0.0391
	*R_L_*	0.6659–0.2851	0.6581–0.2779	0.6729–0.2915	0.5608–0.2034
	*R* ^2^	0.9895	0.9764	0.9714	0.9774
Freundlich	*n*	1.4102	1.4098	1.2472	1.5248
	*K_F_* ((mg/g)(mg/L)^−1/*n*^)	2.115	2.284	2.886	3.185
	R^2^	0.9904	0.9907	0.9908	0.9889
Temkin	*B_T_* (J/mol)	10.88	11.28	18.56	11.22
	*K_T_* (L/mg)	0.3003	0.3193	0.1706	0.4301
	*R* ^2^	0.9844	0.9771	0.9520	0.9786
Dubinin-Radushkevich	*q_max,D-R_* (mg/g)	23.50	24.23	30.66	25.92
	*K_D-R_* (mol^2^/J^2^)	8 × 10^−6^	7 × 10^−6^	4 × 10^−6^	5 × 10^−6^
	*E* (kJ/mol)	0.250	0.267	0.353	0.316
	*R* ^2^	0.8736	0.8631	0.9049	0.8808

**Table 4:** Thermodynamic parameters for the adsorption of CR onto the cross-linked C/CRPW composites and CRPW.

Thermodynamic Parameters	T (K)	1.75C/0.25CRPW	1.50C/0.50CRPW	1.25C/0.75CRPW	CRPW
Δ*G*° (J/mol)	298.15	1052.27	668.89	−878.22	177.40
	308.15	199.92	−164.92	−1691.12	−171.24
	318.15	−652.43	−998.73	−2504.01	−519.88
	328.15	−1504.78	−1832.54	−3316.90	−868.51
Δ*H*° (kJ/mol)		26.465	25.528	23.358	10.572
Δ*S*° (J/mol·K)		85.235	83.38	81.29	34.86

**Table 5:** Comparison of maximum adsorption capacities of different adsorbents obtained by different agricultural waste and chitosan-based composites for CR dye.

Adsorbent		Adsorption Capacity (mg/g)	Reference
Agricultural Waste	Cabbage waste powder	2.313	[54]
	Activated carbon prepared from coir pith	6.72	[55]
	Bengal gram fruit shell	22.22	[56]
	Coconut-based activated carbon fibers	22.1	[57]
	Chinese yam peel–polypyrrole composites	86.66	[58]
	Tunics of the corm of the saffron	6.2	[59]
	CRPW	36.50	Present work
Chitosan-based Composites	Cellulose/chitosan	40.00	[60]
	DNA-chitosan	12.60	[61]
	Chitosan/*Moringa oleifera* gum	50.25	[62]
	Lignin/chitosan	173.0	[63]
	1.75C/0.25CRPW	38.92	Present work
	1.50C/0.50CRPW	39.66	Present work
	1.25C/0.75CRPW	43.57	Present work

## Data Availability

The data presented in this study are available on request from the corresponding author.
